# Experimentally increased snow depth affects high Arctic microarthropods inconsistently over two consecutive winters

**DOI:** 10.1038/s41598-022-22591-5

**Published:** 2022-10-27

**Authors:** Eveline J. Krab, Erik J. Lundin, Stephen J. Coulson, Ellen Dorrepaal, Elisabeth J. Cooper

**Affiliations:** 1 Department of Soil and Environment, Swedish University of Agricultural Sciences, 75007 Uppsala, Sweden; 2 Department of Ecology and Environmental Science, Climate Impacts Research Centre, Umeå University, 98107 Abisko, Sweden; 3 Swedish Polar Research Secretariat, Abisko Scientific Research Station, 98107 Abisko, Sweden; 4 SLU Swedish Species Information Centre, Swedish University of Agricultural Sciences, 75007 Uppsala, Sweden; 5 Department of Arctic Biology, University Centre in Svalbard, PO Box 156, 9171 Longyearbyen, Norway; 6 Department of Arctic and Marine Biology, Faculty of Biosciences Fisheries and Economics, UiT-The Arctic University of Norway, 9037 Tromsø, Norway

**Keywords:** Biodiversity, Climate-change ecology, Ecosystem ecology, Climate-change impacts

## Abstract

Climate change induced alterations to winter conditions may affect decomposer organisms controlling the vast carbon stores in northern soils. Soil microarthropods are particularly abundant decomposers in Arctic ecosystems. We studied whether increased snow depth affected microarthropods, and if effects were consistent over two consecutive winters. We sampled Collembola and soil mites from a snow accumulation experiment at Svalbard in early summer and used soil microclimatic data to explore to which aspects of winter climate microarthropods are most sensitive. Community densities differed substantially between years and increased snow depth had inconsistent effects. Deeper snow hardly affected microarthropods in 2015, but decreased densities and altered relative abundances of microarthropods and Collembola species after a milder winter in 2016. Although increased snow depth increased soil temperatures by 3.2 °C throughout the snow cover periods, the best microclimatic predictors of microarthropod density changes were spring soil temperature and snowmelt day. Our study shows that extrapolation of observations of decomposer responses to altered winter climate conditions to future scenarios should be avoided when communities are only sampled on a single occasion, since effects of longer-term gradual changes in winter climate may be obscured by inter-annual weather variability and natural variability in population sizes.

## Introduction

Snow is one of the most important climatic drivers affecting the Arctic environment and its biology^1,2^. As Arctic soils are an immense global carbon (C) store, it is important to understand how changes in snowfall and snow cover duration occurring under current climate change^3^ affect soil biota that could release large parts of this C to the atmosphere. Microarthropod soil fauna (i.e. mites (Acariformes) and Collembola) are important decomposers driving C release from soils by affecting nutrient cycling and microbial decomposers^4,5^. In Arctic ecosystems, microarthropods form a highly diverse group of soil biota that can occur in great densities reaching well over 100,000 individuals per m^2^ e.g.^6,7^. Despite their great abundance, microarthropod occurrence and activities are largely constrained by low temperatures^8^. Ongoing winter climate change, such as changes in snowfall magnitude and timing, may thus significantly affect these communities and consequently ecosystem functioning.

Microarthropod communities have been reported to respond to various aspects of winter climate change. Studies that manipulated snow depth^9–11^, induced extreme temperature fluctuations^12^, increased freeze–thaw cycles^13,14^ induced ice encasement^15^ and the timing of snowmelt^16^ showed that in many cases microarthropods are negatively affected by experimentally altered snow conditions. Winter climate change effects on microarthropod community composition may occur because microarthropods differ in sensitivity to changes in abiotic factors^12,17^. For example, taxa with low cuticular permeability, small body size and low dispersal abilities (e.g. deeper living Collembola and juvenile mites) are most sensitive to changes in soil temperature and moisture^12,18,19^, and most likely respond strongly to soil microclimatic changes as a result of altered snow patterning. Conversely, larger surface dwelling Collembola particularly adapted to living in snowy environments^20,21^ could e.g. be expected to respond less strongly or in an opposite way to changes in snow packs resulting from winter climate change. Changes in snow cover may thus result in changes in microarthropod densities and/or species-specific responses that may be meaningful for the soil processes microarthropods contribute to Ref.^19^. Therefore, an understanding of microarthropod community changes to altered winter climate is required.

Although winter climate changes may have predictable effects on microarthropod communities based on their species’ characteristics e.g.^12^, many winter climate manipulation studies show inconsistent or unpredictable community responses (e.g.^22^) and that in many cases communities do not respond strongly at all^23,24^. A reason for this discrepancy may be that various effects of changes in snow patterning may affect microarthropods in different directions. For example, experimental snow removal studies typically induce extremely low winter soil temperatures, which can kill large parts of the soil community, whereas snow addition potentially has an opposite effect decreasing the occurrence of low extremes. At the same time, despite a potential positive effect of snow addition on soil temperatures, additional snow can prolong the snow-covered period into spring with potential negative consequences for the life history of soil organisms (e.g. lower soil temperatures in spring). Identifying the main mechanisms by which changes in snow patterning affect the microarthropod community may be a first step towards an increased understanding of soil community responses to changes in winter climate.

Subtle changes in mid-winter soil temperatures may have limited effects on microarthropods. Microarthropod strategies avoiding mortality during the coldest periods in winter such as freeze avoidance (e.g. producing anti-freeze proteins) and cryoprotective dehydration^17,25^ are mostly associated with inactive hibernation. Therefore, a few degrees changes in winter soil temperature may have limited effects on these organisms^23,24^ or should be so extreme that they will break their hibernation^12^. Effects of winter climate change are thus likely most pronounced when affecting conditions around the onset and offset of this hibernation. It can be expected that changes in microclimatic soil conditions during the periods around snow appearance and snowmelt, have the greatest effects on microarthropods^16^. Winter climate change may disturb natural timing and magnitudes of snowfall around these periods and thereby the soils’ microclimate and its inhabitants.

Winter climate change-induced alterations in snow patterns greatly affect snowmelt timing and summer onset^1^. This will have large consequences for soil organisms^26^, as the insulating capacity of snow generally decouples soil from air temperatures making winter soil temperatures more stable and greater than the air above^26^. Shortly after snowmelt and topsoil thaw, microclimatic conditions are suitable for the resumption of activity of Arctic microarthropods, making snowmelt date a determining factor for e.g. the timing and quantity of the organism’s reproductive cycling^27,28^ strongly affecting summer community composition^16,29^. Additionally, snowmelt and soil thaw trigger the start of vegetation growth and development (e.g.^30–33^), indirectly affecting microarthropods, as their communities are strongly associated with the rhizosphere^34^ and vegetation patterning^24,35^. Furthermore, snow(melt) plays a major role in shaping local soil moisture conditions that are crucial for soil invertebrates^36^ and especially important in Arctic ecosystems that are generally known to receive little precipitation during the summer^37^. Depending on the topography, increased snow cover will likely induce a prolonged period of high soil moisture conditions in early summer and simultaneously decrease soil temperature and its (diurnal) variability. Snow patterning changes may thus affect summer microarthropod communities via legacy effects on their life-history, via other parts of the food web, and by altering abiotic conditions during the succeeding summer months.

The relationship between specific winter climate changes and community changes may finally be obscured because microarthropod communities are only effectively sampled during the snow-free Arctic summer and thus may reflect an integration of consequences of preceding winter/spring conditions rather than the effect of spring temperature alone. Studies that sampled microarthropod communities in Arctic ecosystems over time are scarce, but show considerable variability in densities over different years^29,38,39^. Natural variability in snow patterning may be a driving force behind these differences in microarthopod densities over years, and at the same time determine the magnitude of the effects of climate change induced changes in snow cover. For example, a deepening of the snow pack of 10 cm may affect soil temperatures in a snow poor winter much more than in a snow-rich winter. In addition, the state of the microarthopod community before the winter season may determine to what extent they are affected by changes in winter conditions. Natural annual variation in winter conditions may thus determine to which extent soil organisms are affected by e.g. extreme events or changes in snowpack^40^.

**Figure 1:**
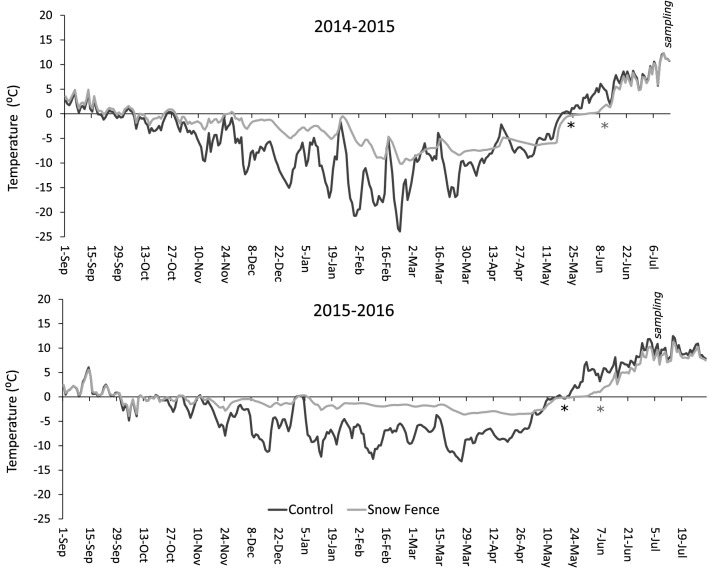
Average soil temperature (1 cm depth) during autumn-early summer in 2014–2015 and 2015–2016 in unmanipulated, ambient (Control, max ~ 35 cm snow) plots and increased snow depth plots (Snow fence, max > 1 m snow) (Number of replicate loggers: 9). Asterisks indicate approximate snowmelt date for ambient (black) and snow fence plots (grey). Experimental site was located at Adventdalen, Svalbard.

To test the effects of increased winter snow cover on microarthropod communities in Arctic tundra and whether these effects were consistent in two different winters in terms of natural snowfall and soil temperatures, we sampled microarthropod communities from a long-term (2006–2016) snow manipulation experiment (fences) in two consecutive summers (2015 and 2016) in the High Arctic valley, Adventdalen, in Svalbard. In this part of the High Arctic, long-term trends show that recent winter temperatures are increasing at a staggering rate of 1.6 °C per decade (vs. ‘only’ 0.2 °C during summers) since the 1960s. There has been an increase in amount of snowfall and snow disappearance is controlled by winter snowfall rather than spring temperature^41^ leading to cooler wetter soils in summer. The snow-fence experiment was established in a heterogeneous landscape naturally differing in soil moisture conditions^42^ and plant species^30^ allowing us to explore whether snow cover effects on microarthropod community composition are consistent geographically across different vegetation types (heath and meadow) and temporally between two consecutive winters. Based on measured soil moisture and temporal patterns in soil temperatures during the 2014–2016 in this snow manipulation experiment (Fig. 1) we postulate the following effects on microarthropod communities:

We expected that effects of increased snow depth on microarthropod density and community composition were overall negative, but variable over the sampled years. More specifically, we expected that in the generally colder winter and spring of 2014–2015, increased snow depth would have a less negative effect on densities relative to its effect in the warmer winter and spring of 2015–2016. At the same time, we expected snow increase to change microarthopod community composition more in 2015 than in 2016. In case of observed community responses, we predicted that given their ecology and morphological characteristics, Collembola would more strongly respond to snow addition than oribatid and predatory mites, and that within Collembola, average community body size would decrease due to species-specific responses to increased snow. Further, we hypothesized that effects of increased snow depth would be stronger in heath vegetation than in meadow vegetation, since meadows are generally associated to a naturally thicker snow cover than heaths. We finally predicted that microarthropod responses to increased snow depth could be mostly linked to changes in abiotic conditions (soil moisture, soil temperature, snow cover) around snowmelt, when temperature and soil moisture conditions drastically change relative to mid-winter conditions.

## Results

In the years investigated, soil temperatures during winter and around snowmelt differed significantly between years. Soils in the winter of 2014–2015 were relatively cold with a mean temperature of − 7.8 °C compared with − 5.0 °C during 2015–2016. This was consistent with air temperatures in the respective years that were somewhat below 10-year averages in 2014–2015, and somewhat higher in 2015–2016 (Fig. S1). Increased snow depth elevated soil temperatures consistently in both years (Fig. 1, Table S1). Mean soil temperatures during winter (October–April) increased by 3.2 °C (from − 7.8 to − 4.6 °C in 2014–2015, and from − 5.1 to − 1.75 °C in 2015–2016). Whereas increased snow depth decreased soil temperatures around snowmelt (May to mid-June) by 2.5 °C in the years (from 0.6 to − 2.1 °C in 2015, and from 2.5 to 0.1 °C in 2016). Increased snow depth even had an impact on temperatures after snowmelt (mid-June to mid-July) when it decreased mean soil temperature by 1.5 °C.

**Figure 2:**
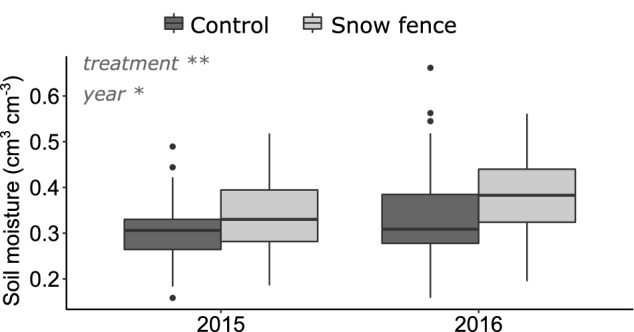
Volumetric soil moisture content (volume water per volume soil sampled) as measured at sampling (early July 2015 and 2016) in unmanipulated, ambient (Control) plots and increased snow depth (Snow fence) plots (n = 11, error bars are standard deviations). Boxes indicate 50% of the central data, the black thick line being the median, black dots are outliers (further than 1.5 times the Box’s range above or below its borders).

Soil moisture during time of microarthropod sampling was greater in plots with increased snow depth and differed between years (Table S1, Fig. 2). There was no effect of vegetation type (heath or meadow) and the effects of increased snow depth on soil moisture conditions were consistent in both years (Table S1). Soil moisture dynamics during the growing season in 2015 show that increased snow depth elevates soil water content during the first weeks after snowmelt, but that this disappeared at time of sampling (Fig. S2). The increased snow depth treatment delayed snowmelt by a mean of 19 and 17 days in 2015 and 2016 respectively (from 20 May to 8 June in 2015, and from 24 May to 10 June in 2016).

**Figure 3:**
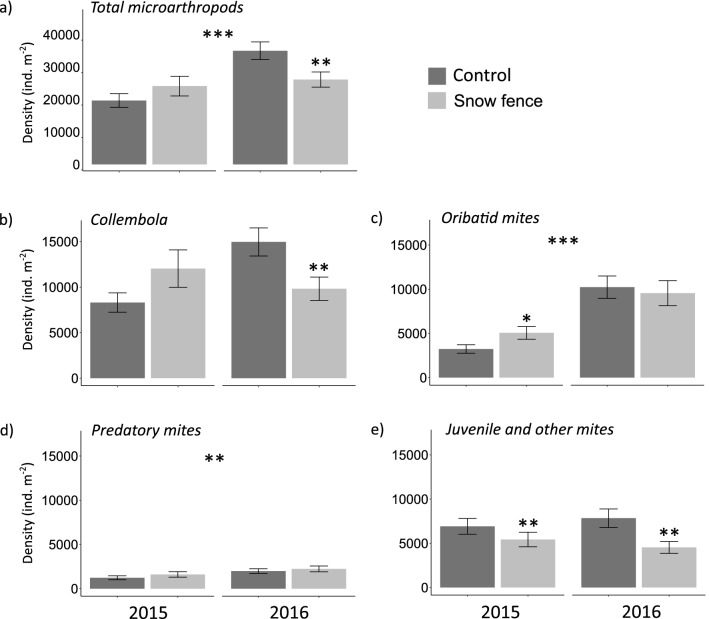
Microarthropod group densities in Arctic tundra (Adventdalen, Svalbard) in experimentally snow accumulated areas behind a snow fence (dark grey) and non-manipulated ambient areas (light grey) sampled in early summer 2015 and 2016. Densities (individuals m^−2^) for (**a**) Total microarthropods, (**b**) Collembola, (**c**) Oribatid mites, (**d**) Predatory mites and (**e**) Juvenile and other mites, are averaged for samples taken from different vegetation types. Asterisks directly above error bars indicate statistically significant difference between treatment effects *within* the respective years. Asterisks in the middle above all bars indicate significant changes in general microarthropod densities over years (n = 11). *P < 0.05, **P < 0.01, ***P < 0.001, error bars are standard errors.

**Figure 4:**
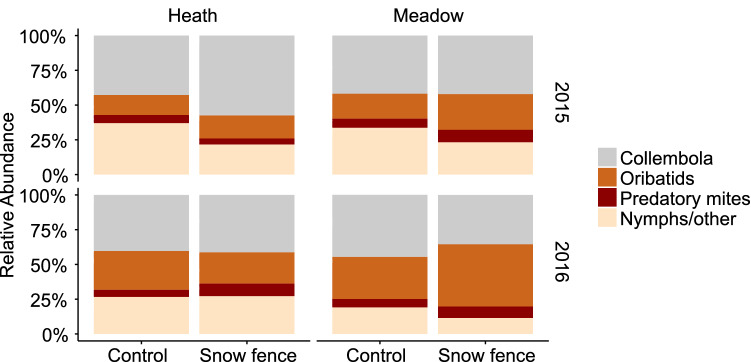
Relative abundances of microarthropod groups in unmanipulated, ambient (Control) plots and increased snow depth (Snow fence) plots in heath and meadow vegetation. Community composition differed between sampling years (***) and was affected by vegetation type (***), but increased snow depth only modestly changed relative abundances and differently over years (*) (n = 6 for meadow and n = 5 for heath plots). *P < 0.05, **P < 0.01, ***P < 0.001.

Microarthropod densities were about 40% greater in early summer 2016 than in 2015 (Fig. 3a, Table S2) coinciding with milder soil temperatures in the winter of 2016, and were not affected by vegetation type (Table S2). Increased snow depth did not affect densities in 2015, but declined densities by 25% in 2016 (Fig. 3a, Table S2). This decline could be attributed to a negative Collembola response (a decline of 34% under snow addition), as they represented the greatest proportion of the microarthropod density (Fig. 3b, Table S2). Oribatid mites were more abundant in early summer 2016 than in 2015, but in contrast to Collembola they positively responded (an increase of 56%) to increased snow depth in 2015, (Fig. 3c, Table S2). Predatory mite density was unresponsive to increased snow depth and showed overall a slightly higher abundance in 2016 than in 2015 (Fig. 3d, Table S2). Mite juveniles and other mites responded negatively to snow depth increase in both years with abundances decreasing 22% in 2015 and 42% in 2016 (Fig. 3e, Table S2). Microarthropod community composition differed between sampling years (F = 11.4***) and was affected by vegetation type (F = 3.0***, Fig. 4, Fig. S3), but increased snow depth only modestly changed relative abundances and differently over years (Fig. 4).

**Figure 5:**
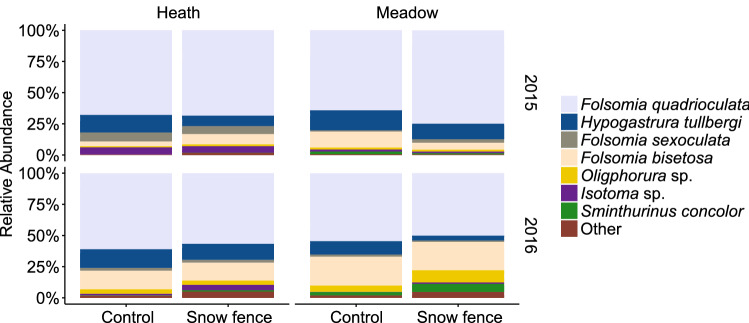
Relative abundances Collembola species or genus in ambient (Control) plots and increased snow depth (Snow fence) plots in heath and meadow vegetation. Collembola species relative abundance differed between years (***) vegetation types (***), and these interacted (**). Snow fence effects were modest, and differed over years (*) (n = 6 for meadow and n = 5 for heath plots). *P < 0.05, **P < 0.01, ***P < 0.001.

**Figure 6:**
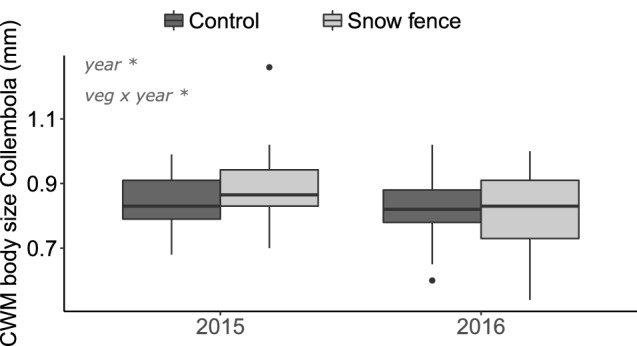
Community weighted means of Collembola body sizes averaged over vegetation types in unmanipulated, ambient (Control) plots and increased snow depth (Snow fence) plots (n = 11). *P < 0.05, **P < 0.01, error bars are standard deviations. Boxes indicate 50% of the central data, the black thick line being the median, black dots are outliers (further than 1.5 times the Box’s range above or below its borders).

Collembola species relative abundance differed between years and vegetation types, and these interacted (F = 3.2***, Fig. 5, Fig. S3). However, relative abundances were only affected by increased snow depth in 2016, but as for microarthropod groups; the changes in relative abundances were modest and without prominent patterns (Fig. 5). The CWM body size of the Collembola was not affected by increased snow depth (Fig. 6), but it was inconsistently related to vegetation type over the 2 years (F = 8.5**), where Collembola in ‘meadow’ vegetation were slightly larger in 2015 than in 2016 (Fig. S4).

Microarthropod density responses can be largely explained by increased snow depth effects on soil temperature from May to mid June (snowmelt period) and the timing of snowmelt (Table S3). Our ‘Covariate models’ replacing the categorical ‘treatment’ factor by the treatment effects themselves (i.e. average winter temperature, spring temperature or snowmelt day) explained up to 36% of the variation, whereas the original ‘Treatment model’ explained 29%. When ‘average winter temperatures’ or ‘early summer temperatures’ were used as factor (both not including the ‘spring’ period), the models performed worse (both explained 21% of variation) (Table S3).

## Discussion

After 9 years of increased snow, its effects on microarthropods were inconsistent between two consecutive winters. Inconsistency in climate responses in Arctic soil invertebrate communities over multiple years has previously been observed in a number of summer warming studies in Arctic tundra and was attributed to inter annual variability in ambient weather conditions e.g.^43–45^. Our observations suggest the same applies for responses to winter climate change.

Experimentally increased snow and its resulting increases in winter soil temperatures did not negatively affect microarthropod densities after the colder winter of 2014–2015, but negatively affected densities after the milder winter of 2015–2016. Snow addition also altered relative microarthropod group abundances in 2016. Unlike observations by Dollery et al.^16^ no relative increase in predatory mites under deeper snow conditions was observed, as microarthropod group changes seemed to mostly be driven by a species wide decrease in Collembola density. However, overall Collembola species composition was affected by experimental snow addition. As Collembola species have different reproduction strategies^46,47^ the effects of increased snow depth, altered snowmelt timing and spring soil temperatures can explain species-specific responses underlying shifts in community composition. For example, *Hypogastrura tullbergi*, is known to adapt its reproductive strategy to seasonality, and has been reported to limit its reproduction when seasons are colder than usual. In contrast, reproduction rate of *Folsomia quadroculata* seems to fluctuate less between years^48^. Indeed, *H. tullbergi* seems to respond somewhat stronger to the snow fence treatment than *F. quadrioculata*, although the differences were rather subtle. Nevertheless, the species-specific changes in Collembola communities observed in 2016, did not seem to lead to distinct ‘functional’ changes, as no changes were observed in mean community body size.

Despite the fact that we only sampled from *Salix polaris* dominated patches, we observed differences in microarthropod composition and Collembola species composition in meadow and heath vegetation, which is in accordance to Bokhorst et al.^49^ that showed the relative abundances of mites and Collembola to differ between heath and meadow vegetation in sub-arctic tundra. Species composition under similar plant species may be highly dependent on the pool of species within the vegetation type and other factors such as soil quality or species interactions^50^. Most interestingly, we observed that Collembola community responses to experimental snow addition differed between vegetation types. However, these did not indicate stronger responses to snow addition from heath-inhabiting communities as hypothesized, but rather of those in meadows. A mechanism could be that vegetation or other (biotic) soil property responses to snow addition may have differed between vegetation types. For example, plant phenology^30^, leaf nutrient status^42^ and respiration^51^ responses to snowmelt differed in the meadow and heath in this experiment. These responses may have cascaded to the microathropod community mediating its responses to snow accumulation.

Although our snow accumulation treatment increased soil temperatures from early autumn up to the date of sampling in early summer in both years, of these microclimatic changes, soil temperatures in the period around snowmelt and the day of snow disappearance seemed to be the best predictors of microarthropod density responses. Significant microclimatic changes during mid-winter and temperatures in the snow-free weeks prior to sampling were worse predictors for microarthropod abundance. This was in accordance with previous studies^16,29,52^ showing that snowmelt timing has great impacts on the microarthropod communities at Arctic and alpine sites. These studies recorded yearly variation in snowmelt date of over 30 days and compared snowbeds to non-snowbeds in which differences in melt-out date are of the same order of magnitude of the observed variation between the years^29,52^. These studies cannot directly be compared to our snow addition experiment due to: the more extreme snowmelt delay (over a month relative to our ~ 17 days), differences in arthropod sampling (pitfall traps), and co-varying differences in vegetation between snowbeds and non-snowbeds. However, they do suggest that in high Arctic areas spring conditions are more important for microarthropod community responses rather than soil conditions in mid-winter. In accordance, Convey et al.^23^ found no difference in overwintering survival of microarthrpods in areas without and with deep snow when arthropods were sampled before snowmelt, suggesting that mid-winter snow conditions may be less important in shaping microarthropod communities than the snowmelt period in high Arctic spring. We propose that snow accumulation interferes in the microarthropod life-cycle, potentially affecting reproduction onsets when snow clears^27,28^ rather than affecting adult mortality in winter.

Instead of directly responding to snowmelt time or soil temperatures in late spring, changes in microarthropod communities may follow changes in soil chemistry or other parts of the foodweb. For example, in an Antarctic arid polar desert experimental snow accumulation induced changes in soil moisture content, pH and nutrient status which coincided with a decline of the dominant nematode species and increase of another^53^. In this snow fence experiment, snow accumulation induced a delay in plant phenology e.g.^30,54^, increased nutrient availability^42,55^ decreased shrub cover^56^, and affected fungal communities^57^. The extent to which microarthropods would respond to changes in fungal diversity in unclear, although microarthropods do have more or less species-specific preferences for certain type of substrates when encountered^58^ they have few problems switching diet^59^. Microarthropods may also respond to changes in plant phenology and nutrient concentrations directly. Unfortunately, none of these previous studies in our site addressed multi-year responses that can be correlated to our study, so it is hard to assess whether trophic cascades may underlie microarthropod responses or the lack thereof.

What could have caused the inconsistency in microarthropod responses to snow depth increase between the studied years? Firstly, ambient microarthropod summer densities differed between the sampled years. Such variation in arthropod densities is not uncommon to observe in multi-year sampling efforts, and has been mostly attributed to climate variability e.g.^39,60^, although species interactions may also play a role^50^. In our study, summer microarthropod densities were higher in 2016 than in the preceding year, suggesting that communities in fall that were faced with winter-conditions also differed. For example, higher Collembola densities may cause stronger inter and intraspecific competition for resources at a cost of survival under cold-stressed conditions. However, we cannot rule out that these density differences were an artifact of our sampling strategy that differed slightly over the two sampled years, since in 2015 samples were transported before microarthropod extraction. This may have caused soils to be affected by e.g. vibrations during transport, which could have caused mortality among microarthropods and thus lower observed abundances in 2015. Regardless, the initial summer communities exposed to snow addition differed between years, which could explain a difference in response, as some species may be more sensitive to changes in microclimate than others^61^. Secondly, snowmelt-day and soil temperatures in the snowmelt period were good predictors for microarthropod responses to increased snow. Differences between ambient temperatures in winter and spring in 2015 and 2016 could be responsible for the difference in responses between the years. Although snowmelt day and the magnitude of the decrease in average soil temperature due to additional snow in the period around snowmelt (about 2 °C) was similar in both years it is most likely that the difference between 0 and − 2 °C (in 2015) had a less effect on the onset of microarthropod activity than the difference between 0 and 2.5 °C (in 2016). Although some microarthropods are active at sub-zero temperatures^62^ it is most likely that the difference between 0 and 2.5 °C will have a larger effect on their activity than the difference between 0 and − 2 °C^63^. For example the thermal threshold needed to complete an instar for *H. tullbergi* lies very close to 0 °C^28^, indicating that warming above this temperature could have larger effects than sub-zero warming. Other winter microclimatic factors such as the occurrence of freeze–thaw cycles and formation of an ice layers to which microarhropods can be sensitive^13–15^ were not visually observed to be remarkably different between the studied years. Not only difference in winter conditions may have driven arthropod responses; summer temperatures in 2014 and 2015 were not substantially lower or higher than average (Fig. S1), but the summer of 2015 was much wetter than the summer of 2014 (Fig. S6). In addition, it may partly explain milder soil temperatures during the winter of 2015/2016, since wet soils with high thermal masses take longer to change temperature. Therefore, snow may have fallen on warmer soils, keeping soil temperatures higher during the remaining winter potentially extending the active period for arthropods. Finally, the inconsistent responses between years can be a cumulative effect as cumulative effects were observed in this site for soil respiration in 2008 and 2011^64^ and linked to decreasing labile C stocks^65^. Also, as most microarthropods survive winter and can live for several years^27^, they may acclimate to optimize reproduction. However, if the effects would be cumulative we would have expected more striking effects of snow accumulation at the first sampling. Unfortunately, the limitation of our time-series to only 2 years does not allow for pattern-finding in the direction of longer-term responses and if they relate to those in soil respiration and labile C stocks. We suggest that high Arctic microarthropods mostly responded to yearly fluctuations in late-spring conditions around snowmelt rather than an ongoing transition in community composition due to longer-term snow accumulation effects.

Studies considering temporal variation in soil fauna community structure are much needed to understand the consequences of climatic changes to belowground food webs and their impact on soil processes^66^. However, longer-term datasets of soil fauna community dynamics are close to non-existent for cold ecosystems (but see^53,67^). Such datasets are much needed to observe trends beyond natural variation in population sizes. Recent intensification of environmental monitoring and ongoing developments in high-throughput molecular techniques simplify connecting soil fauna community dynamics to inter-annual climatic variation. However, coordinated efforts monitoring soil organisms in the Arctic are still largely lacking, even though inter-annual climatic variations are considered to be the strongest drivers of their community dynamics in these ecosystems^29^. However, the impact of longer-term more gradual climate changes, such as alterations in snowfall patterning, may still be effectively assessed by manipulation experiments when sampling efforts are conducted over multiple years after the onset of manipulation. We acknowledge that studying the effect of a decade of snow accumulation on microarthropod communities in two consecutive years hardly allows for drawing strong conclusions about the importance of inter-annual climate variability. But our observations of the range of responses of the microarthropod community to two seasons with contrasting temperature and precipitation underpin that, especially in extreme ecosystems such as high Arctic tundra, extrapolation from 1-year sampling studies should be avoided. Considering the sensitivity of Arctic C stores to climate change, reliable information about organisms driving C turnover is lacking yet required to increase our understanding of the mechanisms underlying future C losses. We therefore advocate for multi-year sampling schemes in long-term manipulative field experiments, and to expand and initiate new long-term monitoring campaigns of Arctic soil communities in these globally important ecosystems.

## Bullet point summary

- After 10 years of experimentally increased snow, microarthropod community responses to deeper snow were inconsistent over two consecutive years.
- Microarthropod densities were mostly unresponsive to increased snow depth in the first of the assessed years.
- In the second assessed year increased snow pack reduced densities of most microarthropod groups and induced changes in relative species composition (but not body size distribution) of Collembola.
- Soil temperatures in the period around snowmelt and the day of snow disappearance were the best predictors of microarthropod density responses.
- Extrapolation of results to predict microarthropod responses to longer-term climatic changes from a single field season can be invalid and should be avoided.
- We advocate strongly for long-term monitoring of Arctic soil communities.

## Methods

### Field site and experimental setup

The study was conducted in Adventdalen, a valley near Longyearbyen, Svalbard (78° 10′ N, 16° 04′ E). The vegetation type is classified as Prostrate/hemiprostrate dwarf-shrub tundra (CAVM Team, 2003) dominated by *Cassiope tetragona, Dryas octopetala* and *Salix polaris*. Mean air temperature 2007 to 2016 at Longyearbyen airport (~ 20 km NW of the study site) from October to April is − 7.5 °C and during the growing season (May–September) 3.8 °C (Fig. S5, www.eklima.no). Mean annual precipitation (2007–2016) was 213 mm of which 132 mm generally falls as snow (Fig. S5, www.eklima.no).

In this tundra, Snow fences that accumulated snow (1.5 m tall and 6.2 m long) and paired ‘ambient’ areas (10–15 m north or south of the snow fences) were established in four experimental blocks in the autumn of 2006. Each experimental block had three fences and ambient areas, except for one that had only two. There were in total 22 plots; 11 manipulated plots with increased snow depth and 11 ambient control plots. Each plot measured 6.2 × 17 m (Fig. S6). Snow depths in ambient plots normally reach a maximum snow depth of ~ 35 cm; whereas snow depth in fenced plots generally range from 60 to 150 cm. Our samples were taken in the plot area where manipulated snow depth reaches ~ 100 cm (within ~ 30 m^2^, more details in Fig. S6). Two of the experimental blocks were established in ‘heath’ vegetation and two blocks in mesic ‘meadow’ vegetation. The dominant vascular plant species in the heath were (in order of dominance): *Cassiope tetragona*, *Dryas octopetala*, *Salix polaris*, *Saxifraga oppositifolia*, *Alopecurus magellanicus* and *Bistorta vivipara*. Mesic meadow vegetation was dominated by *Salix polaris*, *Luzula arcuata* subsp. *confusa, Alopecurus magellanicus*, *Dryas octopetala* and *Bistorta vivipara*^30^.

Each plot had a temperature logger (Tinytag data loggers, model TGP‐402 (Gemini)) placed just below the soil surface (in the increased snow depth treatment where snow depth reaches ~ 150 cm), but data from these loggers was not available for all plots in both years. As some temperature loggers showed a drift in sensor readings (< 20%), observed temperatures were corrected before data analyses by defining the period before snowmelt when soil temperatures are constantly close to 0 for multiple days (the ‘zero curtain’)^51,68^. For some loggers, readings in this period were consistently more than a degree above or below zero thus we corrected year-round temperatures for this deviation. Mean soil temperatures were calculated for different periods of the season for analyses: ‘Seasonal mean for winter’ was defined by the snow cover period (October–April), ‘Seasonal mean temperature around snowmelt’ comprised the period between the first and last experimental plot to become warmer than 0 °C (May–mid-June). ‘Seasonal mean temperature for early summer’ accounted for the time after snowmelt and before sampling (mid-June–mid July). Snowmelt date (day of year) was obtained using the zero-curtain and was defined as the first day the soil temperature exceeded one degree above zero, and defined for each individual plot. Unfortunately, no data on plot-specific snow depth was available.

Soil moisture at time of sampling (in both sampled years) was determined gravimetrically from each plot using three replicate (ø 4.5 cm, 5–9 cm deep) cores from *Salix polaris*-dominated patches that were also used for microarthropod extraction. These cores were weighed upon sampling, subsequently dried at ~ 35 °C for 7 days during microarthropod extraction, and finally dried in an oven at 70 °C for 48 h. Water weight was assumed to correspond to volume, and soil volumes were obtained by measuring the sampled soil depth to determine soil moisture content (water volume/ volume).

Soil moisture dynamics throughout the growing season of 2015 was monitored by using a Theta ML 2X probe (Delta‐T Devices) and expressed water content by percent water. Measurements were made biweekly in areas where maximum snow depth in the increased snow depth treatment is ~ 100 cm and in the respective ambient control plot next to each snow fence.

### Sampling

Three cores were taken (ø 4.5 cm, 5–9 cm deep) from each fence/ambient plot from *Salix polaris*-dominated patches in the early summer of 2015 (15th of July) and 2016 (6th of July). In increased snow depth plots, the cores were taken approximately 10 m west of the snow fence. Cores were taken so that the samples always contained the complete organic layer (on average approx. 5 cm thick (Semenchuk et al.) in which most microarthropods can be found, as well as a part of the mineral soil (> 1 cm), in which microarthropod densities are generally low or absent^69^. In 2015, these cores were stored at 6 °C and transported to Abisko, Sweden for Tullgren extraction (12 bank Tullgren funnel, Burkard Scientific, Uxbridge, UK) within 4 days after sampling. In 2016, the cores were stored overnight at 6 °C and extracted in the same type of Tullgren extractor at UNIS in Longyearbyen the day after sampling. Both Tullgren extractions lasted for 7 days to ensure the cores were completely dry.

### Microarthropod identification and calculations

Microarthropods were identified to ‘group level’, ‘Collembola’, ‘Oribatid mites’, ‘Predatory mites’ (Prostigmata and Mesostigmata) and ‘Mite juveniles/other’ (nymphal Oribatids and Mesostigmata and nymphal Prostigmata) and counted. Collembola were identified to species or genus level^70^, counted and deposited at the Abisko Scientific Research Station, Abisko Sweden. Average body size/length per Collembola species was determined for 30 randomly chosen individuals per species (or as many individuals as available, a minimum 10 individuals) by measuring Collembola length in dorsal aspect from the head to tip of the abdomen by a calibrated microscope (Leica, × 40 magnification). The body lengths obtained were used to calculate community weighted mean (CWM) body size of the community. We calculate the CWM for a whole community as:

$${\text{CWM}}_{{\text{j}}} = \sum\limits_{{{\text{k}} = 1}}^{{{\text{n}}_{{\text{j}}} }} {{\text{A}}_{{\text{k,j}}} \times {\text{FT}}_{{\text{k,j}}} } ,$$

where n_j_ is the number of species sampled in community j, A_k,j_ is the relative abundance of species k in community j and FT_k,j_ is the functional trait of interest of species k in community j.

### Statistical analyses

To test how increased snow depth affected soil moisture (at sampling) we used linear mixed models with ‘increased snow depth treatment’, ‘vegetation type’ and ‘year’ as fixed factors and ‘plot nested in block’ as random factor (to account for spatial dependence between blocks and the plot’s three sub-samples) Snow effects on soil temperature was tested by reducing the dataset to only incorporate paired increased snow depth and ambient plots with available logger data for both years. Using this subset we ran three linear models with fixed factors; ‘increased snow depth treatment’ and ‘year’ in which dependents were either ‘average soil temperature from October to April, ‘average soil temperature from May to mid June’ and ‘average soil temperature mid-June to mid-July’.

To assess if increased snow depth affects total microarthropod densities and group-specific densities, we used linear mixed models. ‘Increased snow depth treatment’, ‘vegetation type’ and ‘year’ were fixed factors and ‘plot nested in block’ was a random factor. If there were interactions between ‘increased snow depth treatment’ and ‘year’, separate models were run for each year. Post-hoc Tukey comparisons were used to test specific-effects. A similar approach was used to assess increased snow-driven CWM body size changes of Collembola.

Effects of increased snow depth on microarthropods and Collembola community composition were assessed by performing permutation tests for multifactorial multivariate analysis of variance (Two-way Permanova, no. of permutations: 999) Dissimilarity matrixes were created using the Bray–Curtis index. Fixed factors were ‘increased snow depth treatment’, ‘block’ and ‘year’. Plot was a random factor to account for the nestedness of observations within plots.

To investigate which microclimatic consequences of increased snow depth microarthropods respond to most, we used the previously described reduced dataset in which soil temperature data were available for ambient-treatment pairs and both sampling years. As plots only had one temperature logger, we calculated means for soil moisture and microarthropod densities (sampled in triplicate) to obtain a symmetrical dataset. To test our hypothesis that the period around snowmelt mainly determines the increased snow depth effects of microarthropod communities, we ran a set of linear models and compared how well these models explained variation in total microarthropod density. In the first ‘Treatment model’, fixed factors were ‘increased snow depth treatment’ and ‘year’, comparable to the original linear mixed model excluding ‘vegetation type’ and the random factor. For the following ‘Covariate models’ instead of ‘increased snow depth treatment’ we added ‘soil temperature’ and ‘soil moisture’ as covariates and ‘year’ as a fixed factor. Three different intervals of ‘soil temperature’ were used as covariate in separate models: ‘average soil temperature from October to April, ‘average soil temperature from May to mid June’ and ‘average soil temperature from mid-June to mid-July’. ‘Snowmelt date’ was inferred from soil temperature data and used in the fourth model. All models were run to test effects on total microarthropod densities and adjusted R^2^ values were compared to address the explanatory power of each model.

Normality and heterogeneity of residuals were plotted and checked visually for residual patterning and values were transformed by square root or logarithmic (log x + 1) transformation when needed. All statistical analyses were carried out using R software (v.4.0.1)^71^. Linear mixed models were run using the ‘nlme’ package, post hoc tests using the ‘multcomp’ package, and permanova analyses using the ‘vegan’ package. Because of a slightly unbalanced design, P-values for the linear mixed models were obtained using Kenward–Roger approximation and ‘Type II SS’ using the ‘Anova’-command in the ‘car’ package.

## Supplementary Information

Supplementary Information.

## Data Availability

Raw data used in this publication are available online from the Dryad data repository: 10.5061/dryad.1zcrjdfv6.
