# Supplementary material for: Experimentally increased snow depth affects high Arctic microarthropods inconsistently over two consecutive winters
Source: Sci Rep. 2022 Oct 27;12:18049. doi: 10.1038/s41598-022-22591-5 (PMC9613649; doi:10.1038/s41598-022-22591-5)
Supplement: Supplementary file 1 — Supplementary Information. [file 41598_2022_22591_MOESM1_ESM.pdf]

Supplementary data

Experimentally increased snow depth affects High Arctic microarthropods inconsistently over two consecutive winters

Eveline J. Krab[1,2*] *(0000-0001-8262-0198)*, Erik J. Lundin[3] *(0000-0002-3785-8305)* , Stephen J. Coulson[4,5] *(0000-0003-0935-959X)*, Ellen Dorrepaal *(0000-0002-0523-2471)* [2], Elisabeth J. Cooper[6] *(0000-0002-0634-1282)* .

[1] Swedish University of Agricultural Sciences. Department of Soil and Environment, SE-75007, Uppsala, Sweden

[2] Climate Impacts Research Centre, Department of Ecology and Environmental Science, Umeå University, SE-98107 Abisko, Sweden

[3] Swedish Polar Research Secretariat, Abisko Scientific Research Station, SE-98107, Abisko, Sweden.

[4] SLU Swedish Species Information Centre, Swedish University of Agricultural Sciences, SE-75007, Uppsala, Sweden

[5] Department of Arctic Biology, University Centre in Svalbard, PO Box 156, NO-9171 Longyearbyen, Norway

[6] Department of Arctic and Marine Biology, Faculty of Biosciences Fisheries and Economics, UiT-The Arctic University of Norway, N-9037 Tromsø

* Corresponding author:

Email address: eveline.krab@slu.se / e.j.krab@gmail.com

Tables

Table S1. Results of statistical analyses for soil moisture conditions at time of sampling, and for soil temperature patterns during the sampling year (excluding Mid-July-August). Soil moisture conditions tested using a linear mixed model, with fixed factors ‘snow accumulation treatment’ (S), ‘vegetation type’ (V) and ‘year’ (Y), (n=66) whereas soil temperature was analysed on a reduced dataset (n=24), using a linear model with fixed factors: ‘S’ and ‘Y’. Statistically significant (α = 0.05) effects are indicated in bold font. **= $P < 0.01$*** = $P < 0.001$.

	Snow acc.	***Veg. type***	***Year***	***S x V***	***S x Y***	***V x Y***	***S x V x Y***
Abiotics							
Soil moisture	**F = 7.05****	*F= 1.13*	***F = 6.54**** ***	*F = 0.17*	*F =0.36*	*F = 0.43*	*F = 0.48*
Average soil T (Oct-Apr)	***F = 33.81*****		***F = 24.09*****		*F = 0.00*		
Average soil T (May-mid Jun)	***F = 47.27*****		***F = 30.46*****		*F = 0.19*		
Average soil T (mid Jun-mid Jul)	***F = 12.20****		*F = 0.00*		*F = 0.48*		
Snowmelt day	***F= 53.08*****		***F=0.80***		*F=0.04*		

Table S2. Results of statistical analyses (linear mixed model, analysis of variance) for microarthropod-group densities. Factors were 'Increased snow treatment (S)', 'vegetation type' (V), and 'year' (Y), random factors included are 'site' nested in experimental 'block'. Full models include year, if year interacted with treatment (*P* < 0.1 included), separate models were ran for each year. Data were transformed where needed to fit model assumptions (†indicates square root transformed data, § log (x+1) transformed data). Statistically significant (α = 0.05) effects are indicated in bold font. ' = *P* < 0.1, * = *P* < 0.05, **= *P* < 0.01*** = *P* < 0.001.

	Model	***Snow acc.***	***Veg. type***	***Year***	***S x V***	***S x Y***	***V x Y***	***S x V x Y***
Density								
Microathr.	Full	*F = 0.79*	*F= 0.28*	***F = 12.61*** ***	*F = 0.04*^{s}	***F = 7.21*** **	*F = 1.99*	*F = 1.34*
	2015	*F = 1.43*	*F=0.05*		*F = 0.88*			
	2016	***F = 7.32*** **	*F = 0.59*		*F = 0.54*			
Collembola	Full	*F= 0.24*	*F = 0.00*	*F = 2.60*	*F = 1.77*	***F = 9.10*** **	*F =2.84'*	*F= 0.82*
	2015§	*F = 0.88*	*F = 0.39*		*F = 0.10*			
	2016	***F = 7.47*** **	*F = 0.29*		*F = 0.11*			
Oribatid mites	Full†	*F = 0.79*	*F = 5.91*	***F = 41.0*** ***	*F = 2.38*	*F = 3.38'*	*F =2.66*	*F = 3.19'*
	2015	***F = 4.58*** *	*F = 2.21*		*F = 0.01*			
	2016§	*F = 0.38*	*F = 3.18*		*F = 3.45'*			
Nymphs	Full†	***F = 8.17*** **	*F = 0.34*	*F = 0.02*	*F = 0.91*	*F = 0.71*	*F = 1.61*	*F = 0.01*
Predatory	Full†	*F = 1.00*	*F = 1.02*	***F = 9.08*** **	*F = 0.09*	*F = 0.64*	*F = 0.00*	*F = 0.85*

Table S3. Results and comparison for fit of linear models describing total microarthropod densities. Models were either ran with fixed factors 'increased snow treatment' (S) and 'year' (Y) (Treatment model) or with 'soil moisture' (SM) and 'soil temperature' (ST) as covariates instead of 'snow-accumulation treatment' and 'year' as a fixed factor (Covariate model). Five covariate models were performed, each differing in soil temperature intervals as described in Table 1 as well as Snowmelt day and Degree Day Sum (Sept- mid-July). When effects differed between years and covariates explained a substantial part of the variation (P < 0.1) we tested for an interaction using a reduced model. Adjusted R^2 values indicate model fit, a higher value represents a better fit. All models were performed on the reduced dataset (N=24). Statistically significant (α = 0.05) effects are indicated in bold font. ' = *P* < 0.1, * = *P* < 0.05, **= *P* < 0.01*** = *P* < 0.001

	Model	*Snow acc*	*Soil moist.*	*Covariate*	*Year*	*Interact. Y*	*Adjusted R^2*
Total microarthropods							
Treatment model	*Full*	*F=1.91*			***F=8.75****	*F=1.76*	*0.29*
Covariate models							
Average soil T Oct-Apr (winter)	*Full*		*F=0.00*	*F=0.88*	***F=8.1****		*0.21*
Average soil T May-mid Jun	*Full*		*F=8.01**	*F=0.07*	*F=3.46'*		*0.27*
(spring)	*Reduced*			***F=9.11****	*F=2.96*	*F=3.8'*	*0.36*
Average soil T mid Jun-mid Jul (early summer)	*Full*		*F=0.23*	*F=0.50*	*F=8.4*		*0.21*
Snowmelt day	*Full*		*F=0.28*	*F=3.93'*	*F=8.00'*		*0.29*
	Reduced			***F=4.41****	***F=8.5****	*F=3.14'*	*0.36*

Figures

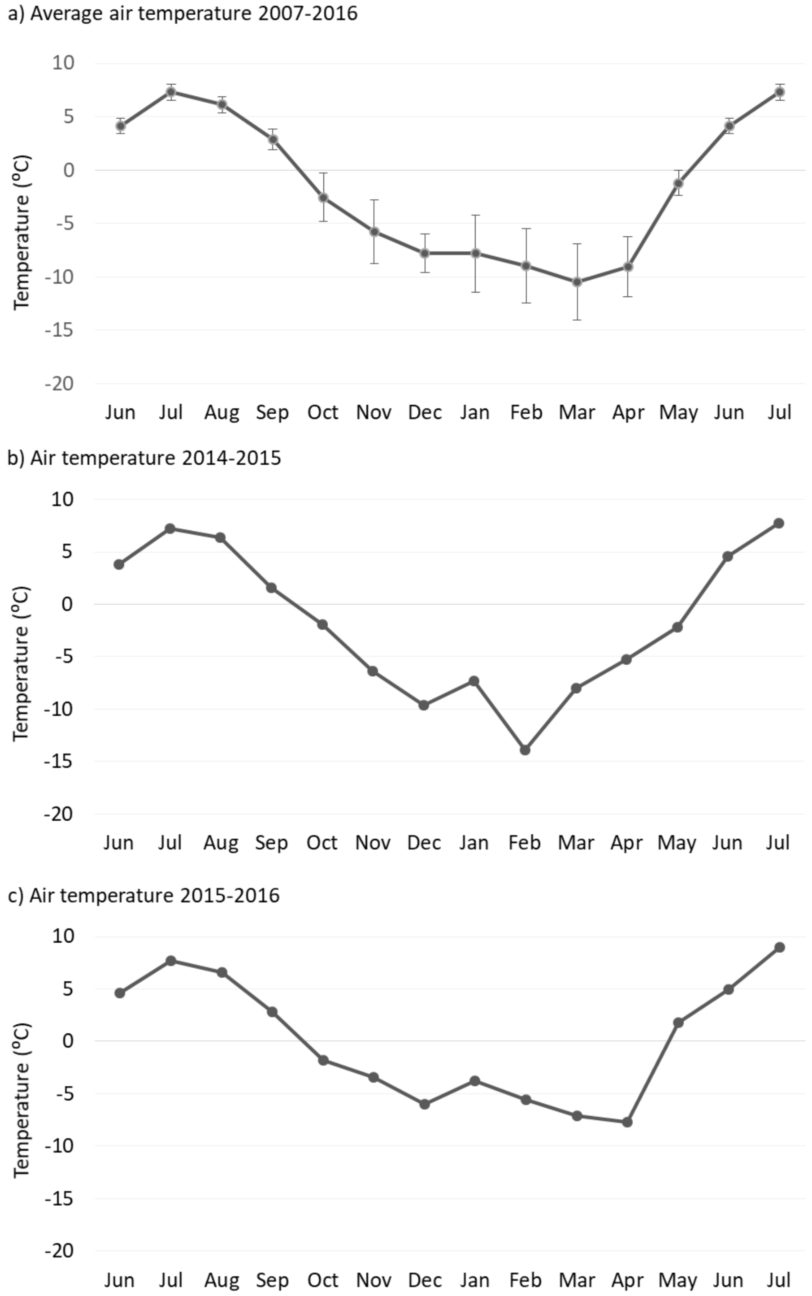

Figure S1. Monthly air temperature: a) 10-year average (2007-2016), b) during sampling year 1 (2014-2015) and c) during sampling year 2 (2015-2016) at Svalbard Longyearbyen airport approximately 20 km from Adventdalen in the same valley (Norwegian meteorological institute, www.eklima.no). Error bars are standard errors.

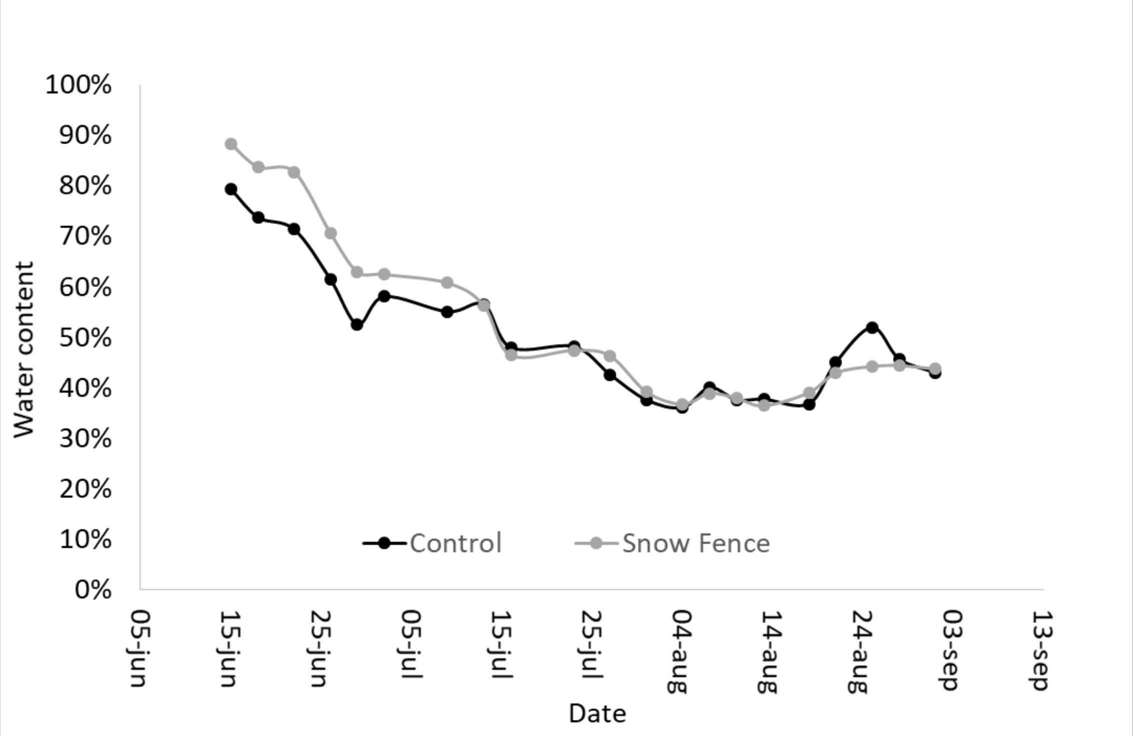

Figure S2. Soil moisture dynamics during the growing season in plots with increased snow accumulation (Fence) and unmanipulated, ambient (Control) areas (n=70)

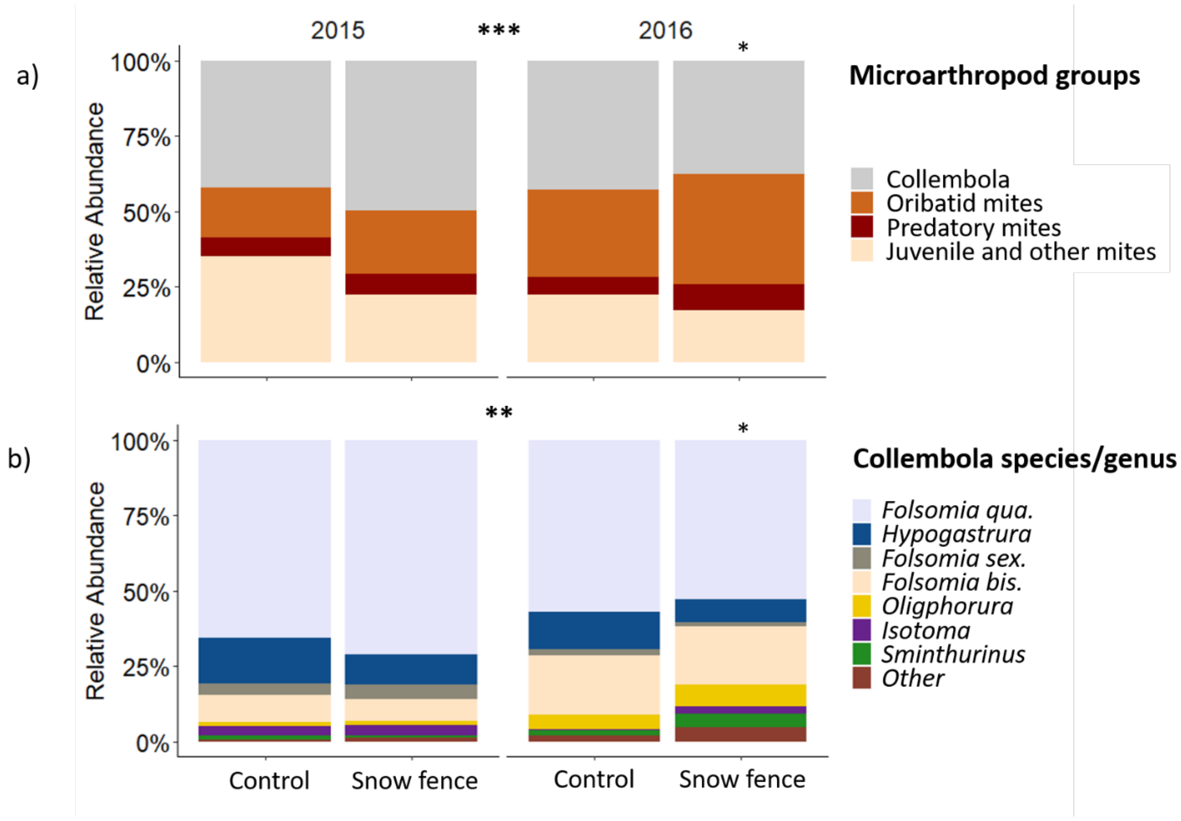

Figure S3. Relative abundances of a) Microarthropod groups and b) Collembola species or genus averaged over vegetation types in unmanipulated, ambient (Control) plots and increased snow (Snow

fence) plots. Asterisks between bars indicate significant effect of sampling year, asterisks directly above bars indicate significant effects of increased snow on community composition (Bray-Curtis distances) of microarthropod groups or Collembola species (indicated by Permanova analyses of separate years). (n=66). * = $P < 0.05$, **=$P< 0.01$, *** = $P< 0.001$.

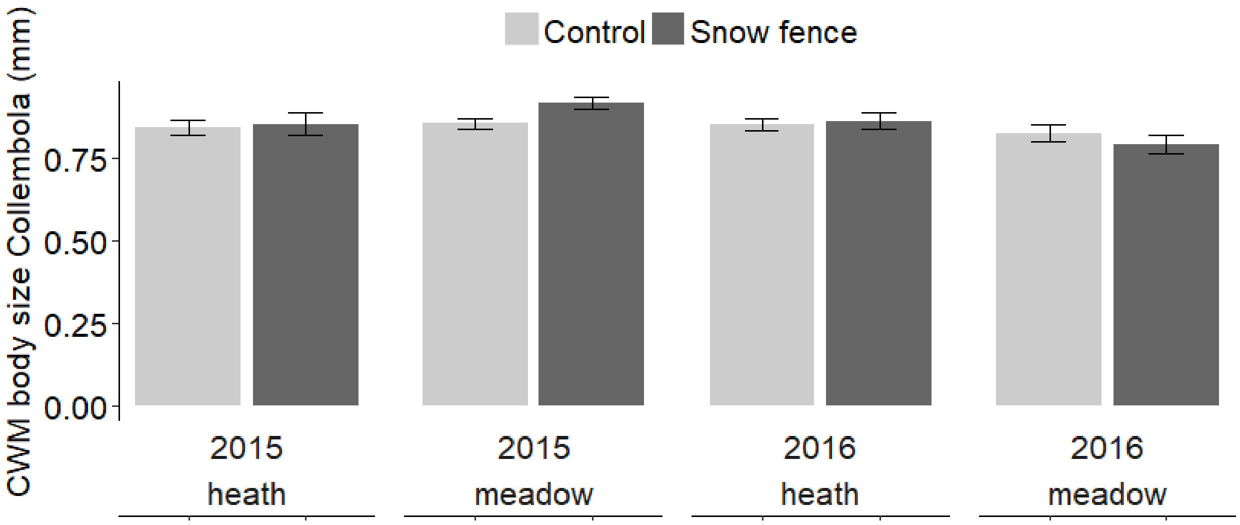

Figure S4. CWM body sizes of Collembola community in unmanipulated, ambient (Control) plots and increased snow accumulation (Snow fence) plots in both years and vegetation types.

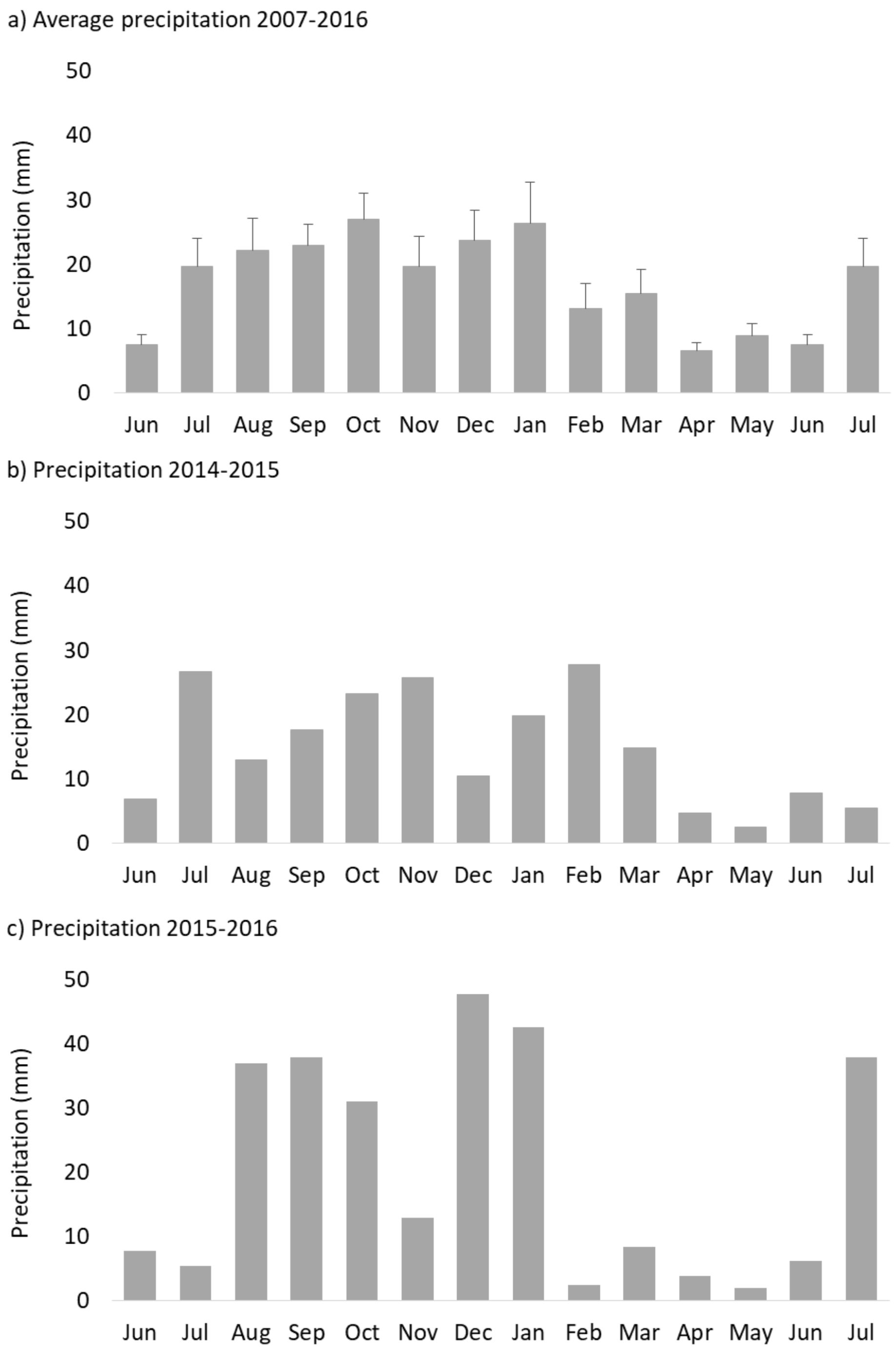

Figure S5. Monthly precipitation a) 10-year average (2007-2016), b) during sampling year 1 (2014-2015) and c) during sampling year 2 (2015-2016) at Svalbard Longyearbyen airport approximately 20 km from Adventdalen in the same valley (Norwegian meteorological institute, www.eklima.no).

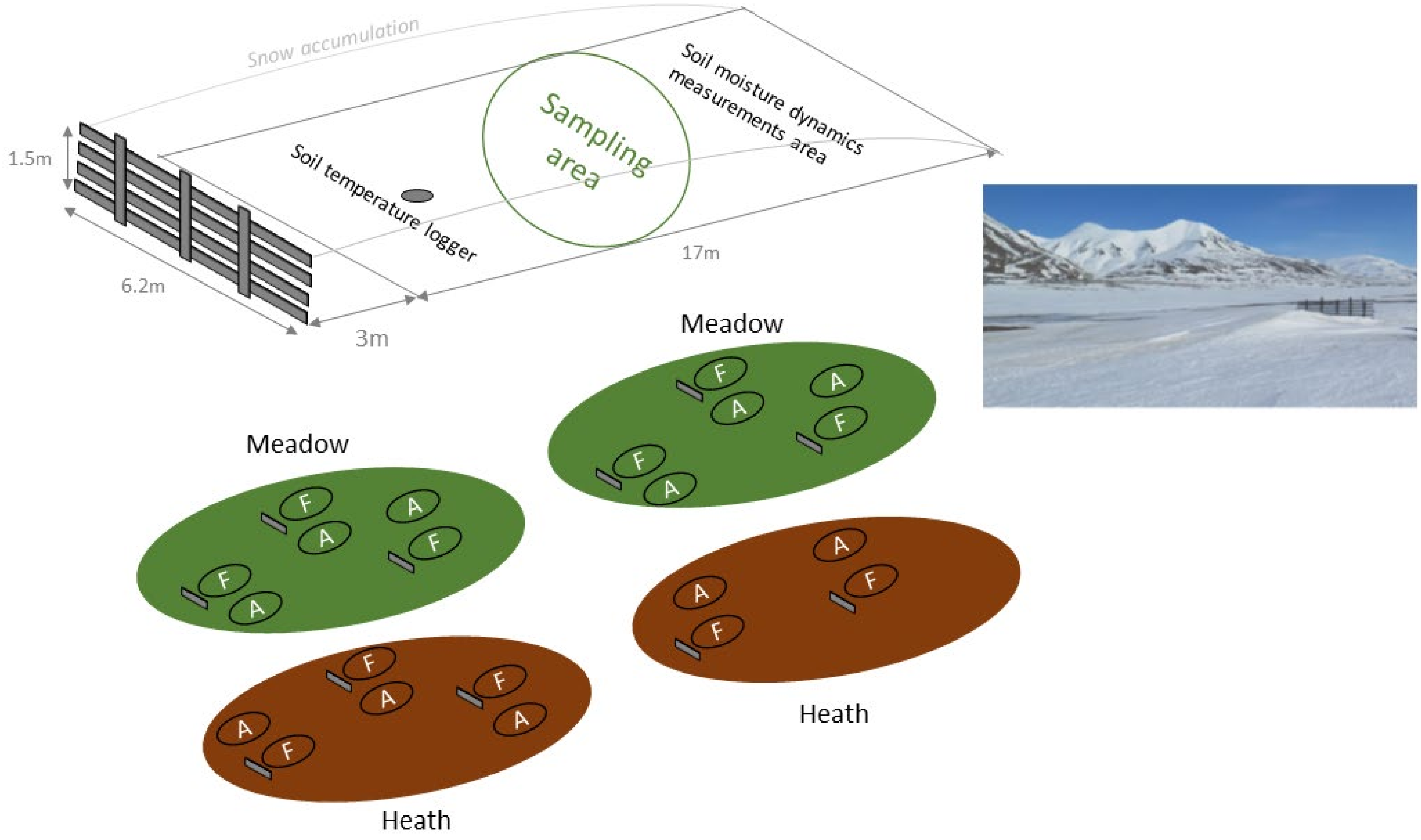
Snow accumulation
Soil moisture dynamics measurements area
Sampling area
1.5m
Soil temperature logger
6.2m
17m
3m
Meadow
Meadow
Heath
Heath
F
A

Figure S6. Experimental setup of experimental plots and block design. Increased snow accumulation plots were approximately 6.2 x 17 m, placed 3-20 m west of each fence or a similar size in the paired ambient area Snow depths in ambient plots reach a maximum of roughly 35 cm; whereas the snow depth in fenced plots generally range from 150- 60 cm. The maximum snow depth is achieved 4-12m behind the fences. The minimum depth is occurs in the north-westerly edge of the plots (~20 m). The snow drifts shapes and extents created by the snow fences are similar each year due to the prevailing easterly wind direction along the valley. Samples were taken ~10 m northwest of each snow fence were manipulated snow depth generally reaches 1 m during the snow-covered period. In the schematic overview of experimental blocks, circles with 'F' represent increased snow accumulation plots, circles with 'A' represent ambient 'control' areas. Ambient areas were unaffected by the snow accumulation fences. Plots were located in arctic tundra vegetation of which two blocks were identified as 'meadow' and two as 'heath'. Picture from Cooper et al. 2019.
